# Non-Invasive Brain Stimulation to Enhance Upper Limb Motor Practice Poststroke: A Model for Selection of Cortical Site

**DOI:** 10.3389/fneur.2017.00224

**Published:** 2017-05-29

**Authors:** Michelle L. Harris-Love, Rachael M. Harrington

**Affiliations:** ^1^Bioengineering Department, Volgenau School of Engineering, George Mason University, Fairfax, VA, United States; ^2^MedStar National Rehabilitation Hospital, Washington, DC, United States; ^3^Interdisciplinary Program in Neuroscience, Georgetown University, Washington, DC, United States

**Keywords:** stroke, transcranial magnetic stimulation, motor cortex, rehabilitation, upper extremity

## Abstract

Motor practice is an essential part of upper limb motor recovery following stroke. To be effective, it must be intensive with a high number of repetitions. Despite the time and effort required, gains made from practice alone are often relatively limited, and substantial residual impairment remains. Using non-invasive brain stimulation to modulate cortical excitability prior to practice could enhance the effects of practice and provide greater returns on the investment of time and effort. However, determining which cortical area to target is not trivial. The implications of relevant conceptual frameworks such as Interhemispheric Competition and Bimodal Balance Recovery are discussed. In addition, we introduce the STAC (Structural reserve, Task Attributes, Connectivity) framework, which incorporates patient-, site-, and task-specific factors. An example is provided of how this framework can assist in selecting a cortical region to target for priming prior to reaching practice poststroke. We suggest that this expanded patient-, site-, and task-specific approach provides a useful model for guiding the development of more successful approaches to neuromodulation for enhancing motor recovery after stroke.

## Poststroke Arm Impairment

Upper limb motor impairment following stroke is highly prevalent and often persists even after intensive rehabilitation efforts (1–4). It is also one of the most disabling of stroke sequela, limiting functional independence and precluding return to work and other roles (5).

Upper extremity motor control relies heavily on input transmitted *via* the corticospinal tract (CST). The CST descends through the posterior limb of the internal capsule, an area vulnerable to middle cerebral artery stroke and in which CST fibers are densely packed. Thus, even a small lesion in this location can have devastating effects on motor function (6–9). A loss of voluntary wrist and finger extension is particularly common and appears to be related to the extent of CST damage (10). There is also evidence that those who retain wrist extension and have considerable CST sparing are more likely to be responsive to existing therapies (7, 8, 11).

However, even individuals who lack voluntary wrist and finger extension often retain some ability to move the shoulder and elbow. Unfortunately, only a few stereotyped movement patterns can be performed and these are often not functional. The combination of shoulder flexion with elbow extension that is required for most functional reaching tasks, for example, is frequently lost. Nevertheless, previous studies have demonstrated that reaching practice with trunk restraint can improve unconstrained reaching ability, even in patients who lack wrist and finger extension (12–15). Still, a great deal of time and effort is required and the improvements are relatively small.

## Non-Invasive Brain Stimulation

Non-invasive brain stimulation offers a potential method of enhancing the effects of practice and thus giving patients greater returns on their investment of time and effort. Approaches to non-invasive brain stimulation are rapidly expanding but generally fall into two major categories: transcranial magnetic stimulation (TMS) and transcranial electrical stimulation [TES; see Ref. (16) for overview of non-invasive techniques for neuromodulation]. These modalities are applied to the scalp overlying a specific cortical area that is being targeted. The level of spatial specificity varies depending on many factors including the modality used (TMS is generally more precise than TES), the stimulation intensity (higher intensity results in a more widespread effect), and the architecture of the underlying tissue. The excitability of the underlying pool of neurons can be modulated by varying stimulation parameters such as the frequency and temporal pattern of the stimuli. Therefore, stimulation can be used to temporarily inhibit or facilitate the underlying cortical area for a sustained period of time after the stimulation ends (usually 20–40 min). In this way, non-invasive brain stimulation could be used to “prime” relevant cortical areas before a bout of practice, potentially enhancing the effects of practice. However, there is little guidance for how such cortical sites might be selected and in which direction (inhibition or facilitation) their activity should be modulated. Conceptual models that could offer such guidance are considered below.

## Mechanistic Models to Guide Neuromodulation

### Vicariation

Vicariation refers to an intact cortical area taking on a function previously performed by the lesioned area. The famous neurologist, Hughlings Jackson, suggested that recovery from brain injury may be dependent on the “re-weighting” of intact areas to increase their role in controlling the affected limb (17, 18). The neural reorganization that occurs following stroke could be considered a kind of vicariation, in which areas spared by the lesion take on (or increase their involvement in) functions previously performed by the lesioned area. Perilesional areas, for example, have been shown to undergo this sort of transformation (19–23).

Another possible site for a vicariation-like process is homologous areas of the non-lesioned hemisphere. There is evidence, for example, that the right-hemisphere homolog to Broca’s area could contribute to poststroke recovery from aphasia (24). Interestingly, in the intact motor system, there is evidence of compensation between left and right dorsal premotor cortex (PMd). When rTMS inhibition of left PMd produced no effect on right hand motor performance, functional imaging confirmed suppression of left PMd activity, but also revealed *increased* activation of right PMd, ipsilateral to the moving hand (25). Only when this compensatory ipsilateral activation was also disrupted were decrements in task performance observed. This suggests that ipsilateral PMd increased its contribution to the movement when the function of contralateral PMd was compromised.

Could an analogous process occur following stroke? Perhaps the disruption of function induced by the stroke could trigger a compensatory vicariation-like process in homologous areas of the non-lesioned hemisphere. This possibility remains controversial, however, as it is difficult to rectify with the many studies that have demonstrated a *negative* relationship between non-lesioned hemisphere activation and motor recovery (26–29).

### Interhemispheric Competition

The notion that non-lesioned hemisphere activity has a detrimental effect on motor recovery is supported not by a model of interhemispheric compensation, as described above, but of interhemispheric competition. Central to this model is the phenomenon of interhemispheric inhibition. Homologous primary motor cortex (M1) representations are interconnected *via* dense transcallosal projections that have primarily inhibitory effects. Thus, a motor representation in one hemisphere tends to inhibit its contralateral homolog, and vice versa, in a relatively balanced manner (30, 31). The interhemispheric competition framework suggests that the negative relationship between non-lesioned hemisphere activation and motor recovery observed poststroke is due to an imbalance in interhemispheric inhibition (32). Damage induced by the stroke is thought to result in a *loss* of interhemispheric inhibition from the lesioned to the non-lesioned hemisphere. Because of this disinhibition, the non-lesioned hemisphere is thought to have abnormally high excitability; which in turn results in stronger interhemispheric inhibition of the lesioned hemisphere. Numerous intervention strategies focused on “rebalancing” this interhemispheric disparity (e.g., decreasing excitability of the non-lesioned hemisphere and/or increasing excitability of the lesioned hemisphere) have been tested, with mixed results (33–38).

Although this model has proven useful for guiding initial approaches to neuromodulation for motor priming poststroke, it does have some notable limitations. First, it relies heavily on data from and characteristics of M1. It is not clear if or how findings from M1 may apply to the physiological interactions and characteristics of other cortical motor areas. The potential role of the non-lesioned hemisphere in recovery is likely dependent on the site, since it is unlikely that all areas of the non-lesioned hemisphere are over-inhibiting their contralateral counterparts. In the study of bilateral premotor cortices referenced above (25), for example, when premotor activity in one hemisphere was suppressed, the increased activity in the opposite “non-lesioned” hemisphere site did not impair performance, but was shown to be supporting it. Thus, the interhemispheric competition model may be less useful when considering the roles of cortical areas other than M1.

### Bimodal Balance Recovery

The Bimodal Balance Recovery Model (16) holds that the relevance of the interhemispheric competition framework depends on the amount of “structural reserve,” or the proportion of the lesioned hemisphere motor system that remains intact following the stroke. According to this model, for those with enough sparing of the lesioned hemisphere motor system, interhemispheric rebalancing may be the best model for driving recovery. However, in those with little structural reserve in the lesioned hemisphere, it is suggested that vicariation within non-lesioned hemisphere motor areas may provide the best option for supporting recovery (34, 39, 40). The concept of “structural reserve” is likely to be captured largely by CST integrity, and indeed, CST integrity is a strong predictor of response to existing treatments and overall recovery (7, 8, 10, 11, 41).

### Structural reserve, Task Attributes, Connectivity (STAC)

As suggested by the Bimodal Balance Recovery Model, CST integrity is a key consideration in selecting a cortical site to target with neuromodulatory priming prior to practice. There are two additional (and closely related) factors that we suggest should also be considered: (1) the characteristics of the motor task and (2) the projection patterns of the candidate cortical sites.

#### Task Attributes

For maximum benefit to be realized, it is necessary to match the targeted cortical site with not only lesion characteristics but also with the type of task that will be practiced in the subsequent treatment session. The cortical site should be selected by considering the neural network that underlies performance of the task to be practiced. Since the neural network underlying a movement varies with certain attributes of the task, it is necessary to carefully consider the attributes of the treatment task(s) that will be trained in the subsequent practice or treatment session (42–45).

One important task attribute to consider is the limb segments and corresponding muscle groups that are involved. The structural and functional characteristics of projections to, for example, proximal and distal or flexor and extensor muscles, can differ (46–48). Perhaps most notably, there are more direct corticospinal projections from M1 to distal muscles than to proximal muscles of the contralateral arm (49, 50), and M1 more strongly influences distal movements (48). Cortical maps of proximal vs. distal limb segments, while overlapping, are still clearly segregated; and representations for distal muscles are larger (51–53) and produce stronger facilitation (54, 55) than those for proximal muscles. Projections to proximal muscles, on the other hand, are more polysynaptic, with cortical projections often synapsing initially in the brainstem or intermediate zone of the spinal cord. In addition, cortical contributions to proximal arm movements such as reaching, originate from a widespread network that includes M1, premotor cortex, supplemental motor area, posterior parietal cortex, and brainstem nuclei. Cortical inputs to proximal muscles are also less strongly lateralized to the contralateral hemisphere than are those controlling distal movements (56, 57), and proximal arm movements thus receive a greater contribution from the ipsilateral hemisphere (58). Other reports have shown differences in the physiological characteristics of proximal vs. distal representations (59), including the physiological mechanisms by which use-dependent reorganization occurs (60).

Notably, much of what we currently understand about mechanisms of poststroke motor recovery in humans is limited to distal hand muscles. Cortical sites contributing to recovery at more proximal joints and muscles are likely to differ from those that underlie recovery of finger movements, due in large part to differences in the sources of neural input to proximal vs. distal muscles outlined above.

Other task attributes to consider include force, speed, or dexterity requirements; whether unimanual or bimanual task performance is required; the level of visual, haptic, or other sensory feedback processing required; whether there is an action selection component, a need to inhibit certain movements, or to produce a specific sequence of movements or movements that are timed to an external cue; to name a few. The neural networks underlying task performance differ based on these attributes, making them an important consideration when selecting a target site for neuromodulatory priming. Prior to practicing a task meant to improve, for example, stereognosis or haptic perception, one might target somatosensory cortex or other sensory processing areas for priming. Similarly, a task that requires precisely timed or sequential movements might suggest supplementary motor area as a target.

#### Connectivity

It is also necessary to consider the structural and physiological connections of candidate sites. Rather than inhibition or facilitation of the entire lesioned or non-lesioned hemisphere, the unique connectivity patterns and computational roles of specific cortical sites within each hemisphere should be considered. Information about structural connectivity is obtained from such sources as transneuronal tracer studies in animal models [e.g., Ref. (61)] and tractography analyses in humans [e.g., Ref. (62)]. Physiological or “functional” connectivity refers to pathways created through the strengthening of synaptic connections. Information about physiological connectivity can be gained by intracellular stimulation and recording studies in animal models [e.g., Ref. (54, 57)]; TMS and electro- and/or magneto-encephalography recordings [e.g., Ref. (63)], and functional and effective connectivity analyses of BOLD imaging [e.g., Ref. (64)] in humans.

Importantly, the connectivity of a candidate region includes its intra- and inter-hemispheric connectivity with other cortical regions as well as its pattern of projections to peripheral targets. One type of connectivity that is often considered is that of transcallosal connections between M1 representations, as discussed above. Note how an understanding of both structural connectivity (i.e., transcallosal projections linking homologous motor areas) and effective connectivity (i.e., the primarily inhibitory effect of activity within these projections) inform the choice for applying inhibitory or facilitatory neuromodulation to non-lesioned or lesioned hemisphere M1, respectively.

Connectivity analyses of functional neuroimaging data have demonstrated that poststroke impairments are frequently related not only to damage of specific cortical sites but also to disruption of the entire network to which those sites belong (65, 66). Intriguingly, there is emerging evidence that non-invasive priming of lesioned hemisphere M1 prior to rehabilitation treatment can prevent the development of abnormal connectivity and enhance beneficial connectivity (67, 68).

While it may not always be possible to directly affect abnormal connectivity *via* non-invasive priming, it is still important to understand the overall connectivity of the system so that alternative pathways may be identified. If the lesion results in a loss of facilitatory input to a key node in the network, for example, another area with facilitatory synaptic connections with that area may be considered as a possible target for neuromodulation. Similarly, modulation of one node in the network can alter connectivity patterns elsewhere within the network [e.g., Ref. (67)].

In addition to intracortical connections, in the motor system, it is critical to consider the available output pathways through which neural drive could be transmitted to the musculoskeletal system. One such consideration is the degree to which transmissions from a candidate area rely upon the structural integrity of areas affected by the stroke. In other words, to what degree does the candidate site rely upon the lesioned area to transmit the desired output? In the case of severe CST damage, for instance, targeting lesioned hemisphere motor areas may not be beneficial if their influence upon the affected limb must be transmitted *via* the CST. Similarly, if CST from the lesioned hemisphere is severely damaged, targeting the non-lesioned hemisphere to improve the affected arm requires a target site with relatively strong projections to the ipsilateral (affected) limb.

In summary, we suggest three main considerations in selecting a cortical site for neuromodulation prior to motor practice: Structural reserve, Task Attributes, Connectivity (STAC). For structural sparing, the key factor seems to be CST integrity; which is reflected, among other things, in voluntary wrist and finger extension. The next consideration is the attributes of the task(s) to be practiced during the subsequent activity-based session. This is important because the neural networks that contribute to the task are determined by specific task attributes. Finally, it is necessary to consider the network connections (both structural and functional) of the candidate sites, depending on the pathway we are attempting to influence.

## Putting it all Together: A Cortical Perturbation Study

Using the STAC framework, we have examined the roles of different candidate brain areas in the control of goal-directed reaching movements performed by chronic stroke patients. The following provides an overview of how the STAC approach was applied and initial results from this ongoing investigation.

### Structural Sparing

According to the Bimodal Balance Recovery model, in patients with greater structural reserve, the lesioned hemisphere would be the primary contributor to affected arm movement, while those with less structural reserve would rely more on the non-lesioned hemisphere (16). Structural reserve is probably best defined as the integrity of the CST. Studies in non-human primates have demonstrated that CST transection primarily results in impaired finger movements (69–71). Therefore, we defined “good” vs. “poor” structural reserve as the presence or absence, respectively, of voluntary finger extension. The presence of good vs. poor structural reserve, then, would point toward the lesioned and non-lesioned hemisphere, respectively, as possible contributors to recovery and potential sites for neuromodulation.

### Task Attributes

In this case, the task is forward reaching. Key attributes of this task are that it is a targeted, goal-directed movement, performed unilaterally using proximal arm muscles and under visual guidance. Visually guided movements to specific locations in the extrapersonal space are known to involve dorsal stream structures, such as PMd (72, 73). Similarly, while M1 projects strongly to distal muscles, PMd has stronger projections to proximal arm muscles (46–48, 70, 71, 74–76). These task attributes raise the possibility of PMd as a potentially important area to target.

### Connectivity of Candidate Areas

Cortical projections to proximal arm muscles originate from multiple cortical motor areas and are often bilaterally organized, in many cases, by way of synapses with brainstem motor areas (77). PMd, in particular, projects to brainstem areas involved in the coordination of visually guided reaching (78, 79). Functionally, PMd is involved in the control of visually guided reaching movements of the ipsilateral arm (57). And, as described above, there is evidence that left and right PMd can compensate for one another, and that PMd can provide necessary input to control the ipsilateral limb (25). While M1 projects most strongly to and is most active during contralateral arm movements, PMd is less strongly lateralized and, therefore, more likely to be able to influence the ipsilateral arm.

Based on these considerations, we predicted that non-lesioned hemisphere PMd would have a greater role in reaching movements of patients with poor vs. those with good structural reserve. We also anticipated that in patients with poor structural reserve, non-lesioned hemisphere PMd would have a larger role than non-lesioned hemisphere M1, given the difference in their connectivity patterns (M1: lateralized projections to distal muscles; PMd: projections to proximal arm muscles and projections to ipsilateral arm).

Patients performed a seated, visually guided reaching task to a flat contact sensor placed on a table at 80% of their maximum forward reaching distance [described in detail elsewhere, (15, 80)]. In response to a visual cue, patients were instructed to “quickly reach out to contact the target.” Notably, the target could be contacted with any part of the hand or fist; no finger movement was required. At varying intervals following the visual cue but prior to movement onset, TMS pulses (two pulses with 25 msec inter-stimulus interval) were delivered to the scalp overlying the targeted cortical site. This stimulation produced a momentary perturbation of the neural processing occurring in that area. If the targeted area was performing a task-relevant computation at the time that the perturbation was applied, this should produce an observable effect on the resulting movement when compared to trials with no perturbation.

**Figure 1:**
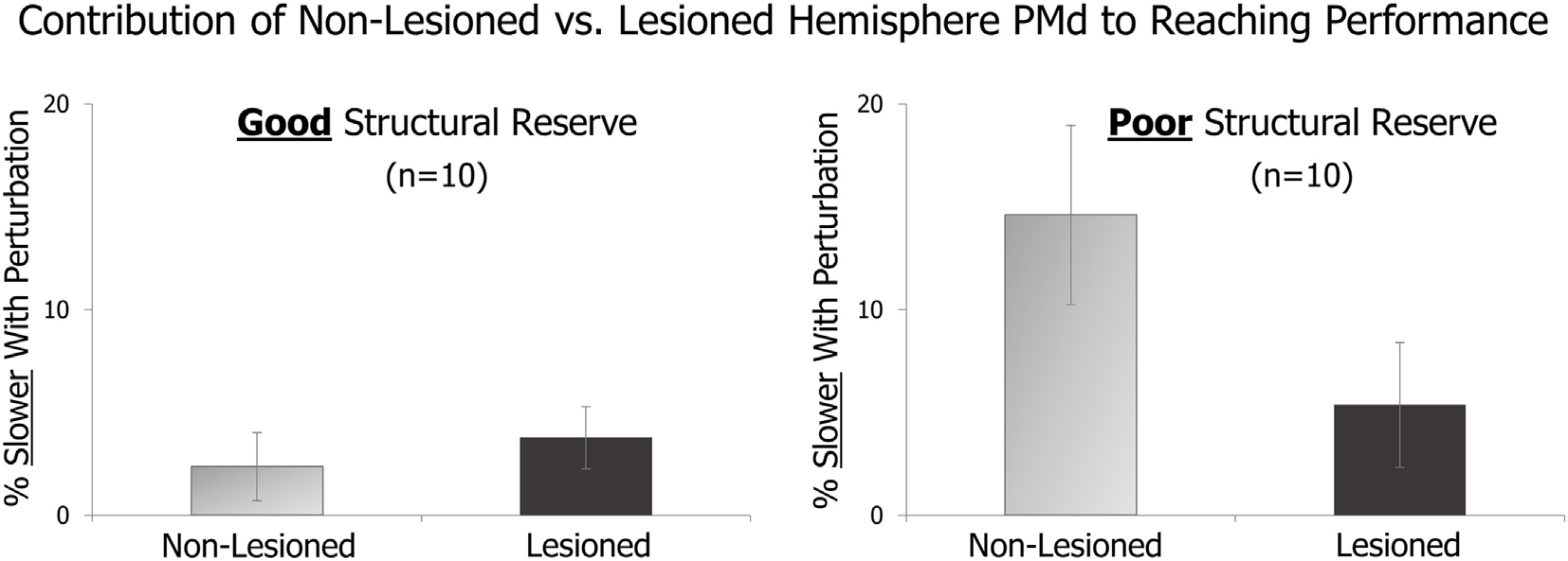
**On randomly delivered trials, transcranial magnetic stimulation (TMS) perturbation was applied just after a “Go” cue**. The effect of this pre-movement perturbation on the speed of the subsequent reaching movement is expressed relative to that in trials with no TMS perturbation. The amount of slowing due to TMS perturbation of the lesioned vs. non-lesioned hemispheres is shown for patients with good structural reserve (left) and patients with poor structural reserve (right).

In an initial analysis, we compared the effects of TMS perturbation applied to lesioned vs. non-lesioned hemisphere PMd in patients with good vs. poor levels of structural reserve. As described above, good vs. poor structural reserve was determined by the presence or absence of voluntary finger extension. Figure 1 displays preliminary results from 10 patients with good structural reserve and 10 with poor structural reserve.

Comparing the light gray bars of the figure, it appears that perturbation of non-lesioned hemisphere PMd had a larger effect on patients with poor (right side) vs. good (left side) structural reserve. This suggests that non-lesioned hemisphere PMd may have a larger role in the movements of those with poorer structural reserve. Similarly, within patients with poor structural reserve (right side of figure), the role of non-lesioned hemisphere PMd (light gray bar) appears greater than that of lesioned hemisphere PMd (dark gray bar).

Additionally, the effects of this perturbation do not appear to be generalized across the entire cortical hemisphere. Instead, the effect appears to differ depending on which cortical site within the hemisphere is targeted.

**Figure 2:**
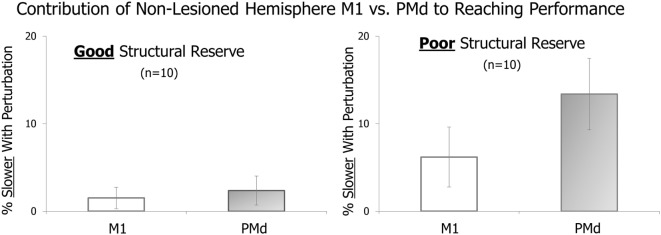
**Effect of pre-movement cortical perturbation using transcranial magnetic stimulation (TMS) on the speed of the subsequent reaching movement**. The amount of movement slowing with perturbation of non-lesioned hemisphere primary motor cortex (M1) vs. dorsal premotor cortex (PMd) is shown for patients with good structural reserve (left) and those with poor structural reserve (right).

In Figure 2, a comparison between cortical sites within the same hemisphere (M1 vs. PMd) appears indicative of a difference in the amount of movement slowing that occurred with perturbation of each site (M1 vs. PMd) in patients with poor structural reserve (right side of figure). Specifically, the movement appears to be influenced more when PMd is targeted than when M1 is targeted, in line with our prediction.

The well-known correlation between non-lesioned hemisphere activation and level of motor recovery does not provide information about the functional role, if any, of that activation. In contrast, directly perturbing the sites of interest, as we have done here, provides causal evidence for their involvement in this task. Using a similar approach, previous studies demonstrated a functional role of non-lesioned hemisphere PMd in affected hand movements (20, 81). The data shown here suggest that this area may also have a role (1) in more severely impaired patients who lack hand movement, (2) in the performance of a multi-joint reaching task that requires activation of proximal muscle groups, and (3) that differs depending on the severity of CST damage. Thus, using the STAC approach and these preliminary findings, one might choose to target non-lesioned hemisphere PMd for motor priming prior to practice of a reaching task in patients with poor structural reserve. Importantly, priming of a different site would be indicated for a different type of task practice in patients with a higher level of structural reserve.

## Summary and Recommendations

To move poststroke neuromodulation to the next level of development, a more specific model is needed for identifying cortical areas that could contribute to motor recovery and be targeted for motor priming. Thus, in addition to the amount of CST damage (i.e., structural sparing) that has been sustained, we suggest consideration of the attributes of the task to be practiced and the connectivity patterns of the candidate sites. This “structure, task attributes, and connectivity” (STAC) approach provides a patient-, task-, and site-specific framework for identifying a cortical area to be primed prior to practice. Our initial investigations in patients with different levels of structural sparing support the utility and validity of this approach.

## Ethics Statement

This study was carried out in accordance with the recommendations of MedStar Health Research Institute Institutional Review Board with written informed consent from all subjects. All subjects gave written informed consent in accordance with the Declaration of Helsinki. The protocol was approved by the Institutional Review Board.

## Author Contributions

MH-L conceptualized, designed, and wrote this submission, critically revised it, had final approval of the version to be published, and is accountable for all aspects of the work. RH contributed to design of this paper, collected data presented, helped critically revise it, had final approval of the version to be published, and is accountable for all aspects of the work.

## Conflict of Interest Statement

The authors declare that the research was conducted in the absence of any commercial or financial relationships that could be construed as a potential conflict of interest.
